# A Review of the Impact of Smoking on Inhaled Insulin: Would You Stop Smoking if Insulin Can Be Inhaled?

**DOI:** 10.7759/cureus.9364

**Published:** 2020-07-23

**Authors:** Parul Malhotra, Radhika Akku, Thulasi Priya Jayaprakash, Olisaemeka D Ogbue, Safeera Khan

**Affiliations:** 1 Medicine, California Institute of Behavioral Neurosciences & Psychology, Fairfield, USA; 2 Internal Medicine, Punjab Institute of Medical Sciences, Ludhiana, IND; 3 Internal Medicine, California Institute of Behavioral Neurosciences & Psychology, Fairfield, USA

**Keywords:** inhaled insulin, smoking, lung permeability

## Abstract

High prevalence of diabetes and the need for tight glycemic control have been well established. With the invention of inhaled insulin, an alternate route has been explored and shows great promise. Inhaled insulin shows a similar physiologic response to subcutaneous insulin, with a faster onset of action, making it suitable for post-prandial hyperglycemia. This comes as a great relief, especially to those who are hesitant to use multiple injections in a day. Many factors affect insulin absorption, including device, particle size, airway patency. Another essential factor is smoking, which is prevalent among people with diabetes, as is in the non-diabetic population. Smoking increases the absorption of inhaled insulin, but it is not a straight fact, since acute smoking, passive smoking, chronic smoking - all have different effects on inhaled insulin. Furthermore, inhaled insulin is also affected by lung diseases. Most studies that have been conducted have included limited populations, thus questioning their generalisability. The studies from inception till 2020 have shown increased permeability of epithelial with acute smoking, change of epithelial layer back to normal after few weeks of smoking cessation, and reverting to chronic smoker levels with just one to two days of start in smoking. Data also suggests that smoking causes a reduction in insulin sensitivity, which could compensate for its increased absorption. Nicotine causes a decrease in the absorption of subcutaneous insulin, but its effect has not been seen on inhaled insulin. More studies, including diabetic smoker patients, need to be performed to give a specific set of variables. This would also add another reason to encourage smokers to quit smoking.

## Introduction and background

In 1989, Von Mehring and Minkowski identified the pancreas as the site of effect in diabetes mellitus. The identification and extraction of insulin was done by Banting and Best in 1921, which is nearly 100 years ago [1]. This discovery gave a new life to all the diabetic patients who, until then, had a life expectancy of about 1.5-2 years and hence was also referred to as the "miracle-cure" [2]. This success prompted more extensive research on insulin. Many different routes have been explored to increase the efficacy, patient compliance, and drug delivery to the body. Ranging from the intravenous route to subcutaneous injections, the research has now landed us with the possibility of inhaled insulin. Pulmonary insulin is gradually rising as a new approach for the treatment of diabetes mellitus [3]. This route's most significant advantage is the extensive surface area of lungs and the thin absorptive surface [2, 3].

At present, two concepts of pulmonary insulin delivery at relatively advanced stages of development have been investigated in several published studies. The first involves a system consisting of a formulation of insulin in a dry and amorphous powder, which is then packaged into blisters. A particular delivery system generates a pulse of compressed air, which causes the insulin to form a white fog in a transparent reservoir that can be inhaled by deep breathing. The second approach uses an aqueous formulation of insulin, delivered as an aerosol generated by a unique, microprocessor-controlled, inhalation device [4].

Inhaled insulin not only has a faster onset of action, but it also stays in the body for a longer duration compared to the regular human insulin and insulin lispro s.c. [5, 6]. These characteristics make it suitable for post-prandial use in diabetics. Also, inhaled insulin and smoking, both primarily affect the lung [7]. Hence, the association between the two is undeniable.

Smoking is associated with much higher morbidity and mortality in diabetic patients than the general population, and the absolute numbers are considerable [8]. Smoking considerably increases the risk of all diabetes complications [9]. Almost 17-26% of diabetic patients smoke cigarettes [10]. There are many long-term adverse effects of cigarette smoking [11], and smoking increases the permeability of the alveolar-capillary barrier in both animal models [12], and humans [13]. Smoking hampers subcutaneous absorption of insulin, increasing dosage requirements [14]. Smoking also causes insulin resistance [15].

Various researchers have studied the impact of smoking on diabetes and insulin. A study by Himmelmann et al. reported that the absorption of inhaled insulin via the AERx® insulin diabetes management system (iDMS) was significantly higher in smokers than in non-smokers [9]. The study emphasized on two parameters AUC (area under the exogenous serum insulin curve from 0 to 6 h) and Cmax (maximal serum concentration of insulin). Another study examined the effects of smoking cessation on the pharmacokinetics of inhaled insulin [10]. They showed that before smoking cessation, AUC and Cmax of insulin after inhalation were higher in smokers than in non-smokers. However, within one week of smoking cessation, the AUC and Cmax had decreased significantly and approached that of non-smokers. Still, there have not been enough studies on the impact of smoking on inhaled insulin [7].

Active cigarette smoking is associated with increased permeability of the pulmonary alveolar epithelium, resulting in faster absorption of inhaled drugs such as Exubera® (EXU). Absorption of EXU is increased approximately twice to four times as much in chronic smokers compared with non-smokers. The trials conducted for inhaled insulin have usually included non-smokers. Hence, there is not much data to give smokers the benefit of this newer and more manageable form of insulin delivery. The studies on smokers have been on non-diabetic patients. This article describes the effect of smoking on inhaled insulin in terms of its pharmacokinetics and raises the need for more research on the same.

With this article, we intend to shed light on the fact that inhaled insulin needs more research in smokers since smokers constitute a significant proportion of the population. For inhaled insulin to be successful, this is a vital aspect to be explored. Also, an increased possibility of inhaled insulin would steer the patients towards a smoking-free lifestyle.

## Review

Inhaled insulin

The interest in an alternate route of insulin administration dates back to 1924 when Von Heubner in Amsterdam and Muller in Germany published the first two papers on the inhalational route of insulin [16, 17]. They illustrated the inhaled liquified insulin and its ability to treat raised glucose levels. Then, after a wait of almost 45 years, Wigley et al. showed that nebulization of porcine insulin could increase plasma levels of insulin and hence help the diabetics [18]. In 1983, Moses et al. performed a study (N=25) and demonstrated the efficacy of Insulin administered intranasally as an insulin-bile salt aerosol [19]. It showed that a rise in insulin levels was linear to the administration of the dose, and that raised the possibility of insulin being used as an aerosol. In 1987, two studies further highlighted the validity of the use of inhalation as a route for insulin administration in both adults and children [20, 21]. Research continued to understand the factors affecting the pulmonary route for drugs. In the 2000s, American companies started their production on a larger scale [7]. In 2004, Barnett reviewed and speculated better chances of glycemic control with Exubera [22]. In 2006, Brain et al. analyzed the data and concluded that Exubera is safe in type 1 and 2 diabetics, and highlighted the immunological factors of the same warranting further studies [23]. In 2015, Ledet et al. reviewed the recent advances in inhaled insulin and found Technosphere® insulin (Afrezza®) to be safe and effective in both type1 and type 2 diabetics [24]. Although the Food and Drug Administration (FDA) has approved Afrezza, more studies are needed to show its efficacy in children, smokers, patients with lung diseases and also shed more light on long term side effects by conducting studies of longer duration and including a broader and more generalizable population.

**Figure 1:**
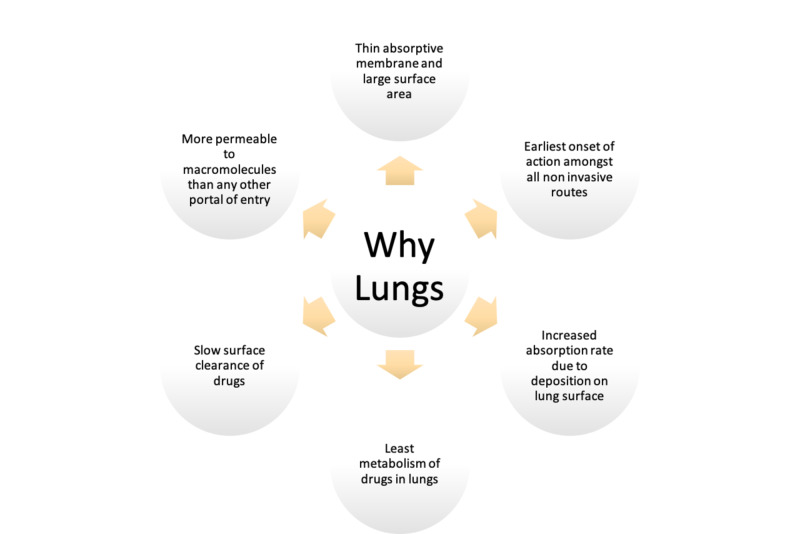
Properties of lungs that make it a good route of drug delivery

The interest has been further increased by the fact that most diabetics receive three to four subcutaneous Insulin injections daily, to which they are not readily receptive, this decreases patient compliance, resulting in difficulties in maintaining glycemic control. Zambanini et al. showed that almost half of the patients missed their injections, and nearly two-thirds of the patients did not want to increase their number of injections due to anxiety [25]. The question was, why were lungs chosen? The lungs have a large surface area and an epithelial lining that makes it more permeable to macromolecules than in any other source [26]. Negligible mucociliary mechanisms in the lungs further promote the uptake of insulin [27]. The motivation to explore this route was also because of the invention of nebulizers for asthma treatment, which highlighted many factors affecting this route of administration and how it can be used for insulin [28]. Figure 1 shows the aspects of the lungs which makes it a good fit for the delivery of drugs.

**Figure 2:**
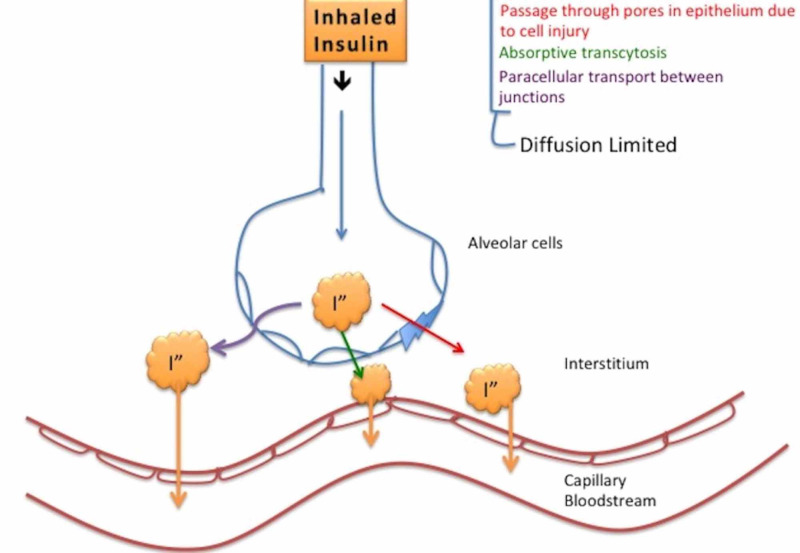
Inhaled insulin absorption mechanisms I" - inhaled insulin

The factors affecting inhaled insulin include particle size, airway geometry, and patency, absorbing surface, efficiency of ventilation, drug formulation, inhalant delivery device. The absorption of inhaled insulin seems to be diffusion-limited [29]. Passey et al. reviewed the articles on inhaled insulin. They postulated that inhaled insulin is deposited by one or a combination of the following processes: impaction, sedimentation, diffusion, and electrostatic precipitation [3]. The Brownian motion may also result due to the interaction of aerosols with gas molecules in the tract [30-32]. After insulin is pumped into the system, 65% of the initial powder within the cartridge reaches the lung [24]. Out of this, 30% is deposited in the oropharynx, 11% is swallowed, and 59% reaches the lung [33]. Unlike the medications for asthma treatment, inhaled insulin utilizes the alveoli more than the rest of the respiratory tract for absorption. Insulin has a molecular weight of 5876 Daltons, and it reaches a peak faster than the subcutaneous insulin and hence is more suitable for post-prandial glucose control and not basal [27]. Figure 2 illustrates the postulated ways of insulin absorption. The absorption is hypothesized to be diffusion-limited and can follow one of the following pathways: through pores in epithelium due to cell injury, transcytosis, and paracellular transport between junctions.

Two main mechanisms proposed are transcellular transport and paracellular transport [26]. Smoking enhances the permeability of the epithelium, hence augmenting the absorption level of inhaled insulin.

Impact of smoking on lungs

Although the awareness has increased and attempts have been made at all levels to reduce smoking, about six million people in the entire world smoke [34], and an estimated 600,000 people lose their lives to second-hand smoke inhalation [35]. Cigarette smoke (CS) consists of two phases, namely the vapor phase and the particulate phase [36]. Earlier estimated to be around 3700, the data now suggests that there are about 4500-4700 particles in CS [35-38]. The majority of these particles damage or modify the lung in several ways, especially the alveolar region, which is also the site of absorption for inhaled insulin. The alveolar-capillary membrane can be broadly divided into the epithelium, interstitial space, and endothelium. The epithelium serves not only as a protective barrier but also has a myriad of other functions such as secreting inflammatory mediators, recruitment of immune cells, maintaining the balance of fluid compartments [39]. The epithelium has many specialized cell types like the ciliated, mucous, goblet, Clara, and basal cells and type 1 and type 2 cells [40]. The cells have tight junctions, which makes them impermeable [35].

**Figure 3:**
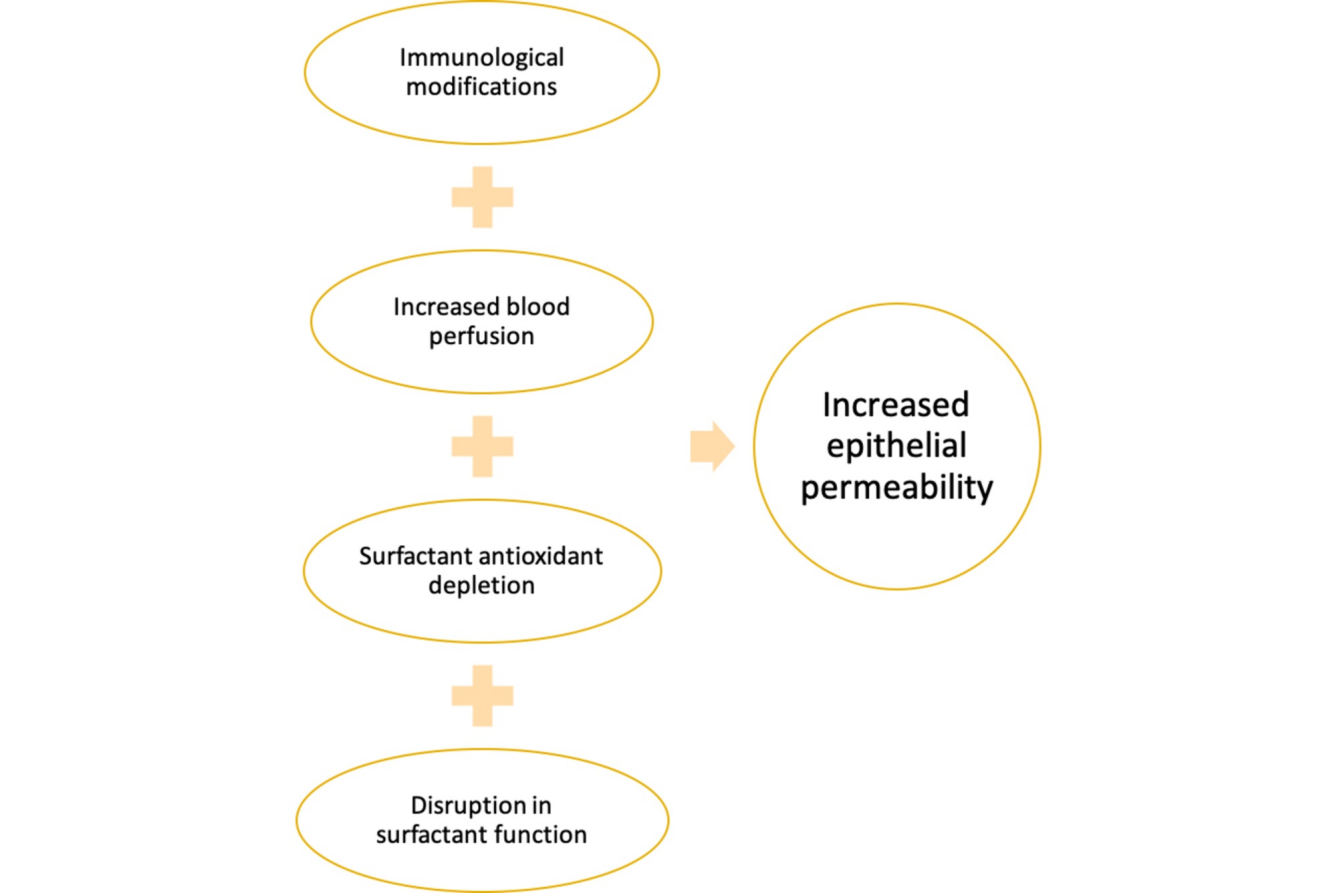
Postulated mechanisms of impact of smoking on the lungs leading to increased permeability of the alveolar epithelial membrane

Jones et al. conducted a pilot study in 1988 where they established that the permeability of the alveolar membrane could be estimated by measuring the transfer of radiolabeled diethylenetriamine penta-acetate (DTPA), and used this method to illustrate that transfer of DTPA. Hence, the permeability of the alveolar membrane was higher in cigarette smokers (p<0.001) [13]. Although it was a pilot study with n=5, it set a good foundation for the concept, steering further research. The mechanism of the increase in permeability by CS could be related to the regulation of adhesion junctions, microtubules [41], and focal adhesion complex [42]. There could be a role of increased oxidative stress in smokers that could cause cell damage and hence increased permeability. CS affects the cells in several ways. CS causes an influx of inflammatory cells, which is responsible for the destruction of the matrix, reduction in blood supply, and loss of epithelial cells [37]. Not only in the lungs, but CS also causes cutaneous vasoconstriction due to the presence of nicotine, which would also reduce the absorption of subcutaneous insulin [36, 43, 44]. This also suggests that not only while prescribing inhaled insulin but also while giving subcutaneous insulin to the patients, the impact of smoking should be considered. Since nicotine reduces subcutaneous insulin absorption, the impact of nicotine replacement therapy (NRT), which is used for smoking cessation, could also impact the insulin absorption in diabetic smokers. However, there is not much data on the same. The increase in permeability of lungs by CS does not have a definite mechanism, but it is nicotine independent [45]. Polycyclic aromatic hydrocarbons, PAH, is one of the significant components in CS, which effects a hepatic cytochrome enzyme, that is responsible for the metabolism of many drugs [36]. Smoking is one of the modifiable risk factors for a majority of lifestyle diseases. Smoking has also been linked to increased morbidity and mortality in diabetics [46]. The following Figure 3, illustrates some of the many ways in which smoking impacts the lungs. Various factors ultimately lead to increased permeability of lungs, which is considered to be the most important factor in alteration of the absorption of inhaled insulin. 

Impact of smoking on inhaled insulin

In 2003, Himmelman et al. conducted one of the first randomized control trial (RCT) to study the effect of smoking on pulmonary insulin delivery [8]. The insulin delivery via the AERx® insulin diabetes management system (iDMS) was studied in healthy smokers and non-smokers. The study concluded that in cigarette smokers, near-physiological insulin profile was attained with AERx iDMS, and absorption of insulin was significantly higher in smokers as evident by the higher area under the curve AUC (0-6 hours) and maximum serum insulin concentration (Cmax) and a shorter Tmax [8]. In another study in 2006, Becker et al. investigated the effect of smoking and its resumption on inhaled insulin and increased insulin absorption in smokers [4]. It was one of the first studies to reveal that smoking cessation for just one week would bring the pulmonary absorption levels to that of healthy non-smoker levels. In contrast, the resumption of smoking after only one to two days could bring the absorption levels to chronic smoker levels. The study also highlighted a secondary finding of a transient change in absorption levels only after 12 hours of smoking cessation, as if acute smoke had an inhibitory effect on absorption initially [4]. However, this fact has not backed by enough evidence or literature and has not been researched. In the same year, Wise et al. conducted a study and concluded that smoking does enhance the absorption of insulin, as seen in previous studies. Still, smoking reduces the glucodynamic (GD) effects of insulin, which could offset its effects due to reduced insulin sensitivity, assessing lung permeability using nebulized insulin [15]. Kohler et al. found that the Cmax of plasma insulin in smokers was between three and four times higher than in non-smokers [47]. In 2007, Pan et al. studied the effect of smoking cessation, acute re-exposure, and nicotine replacement therapy (NRT) on inhaled insulin [48]. Data from this study demonstrate that stopping smoking for four weeks is associated with an evident decrease in AIR insulin exposure and GD response and that the magnitude of this effect is more significant in participants using NRT compared with those not using NRT. Fountaine et al. conducted a cross over study to establish an effect of passive smoke, which was opposite to that of active smoking, according to which passive smoking reduces lung permeability and decreases insulin bioavailability, thereby not posing any risk of hypoglycemia [49]. This fact is also in line with the fact that radioaerosol (DTPA) clearance from the lung decreases in subjects exposed to passive CS, which indicates reduced permeability. The mechanisms behind the same are not entirely understood, although the role of pulmonary blood flow changes and surfactant properties have been advocated [50]. In 2012, Takano et al. conducted an experiment, where they examined the effect of cigarette smoke extract (CSE) on the molecular structure of A549 cells and then its effect on fluorescein isothiocyanate-labeled (FITC) insulin transport [6]. A549 cells are an epithelial cell line derived from human lung carcinoma, and their application in studying various biochemical studies have been used. The experiment demonstrated that CSE directs the pretreated A549 cell lines to type-II phenotype cells, increasing the uptake of insulin and its transport across the epithelial layer. This also supports the findings of earlier studies, stating that inhaled insulin absorption is higher in smokers than in non-smokers. These studies have been summarized as follows in Table 1.

**Table 1:** Summary of reviewed studies AUC - area under the curve; Cmax - maximum serum concentration; Tmax - time to drug peak; RCT - randomized control trial

Author	Study	Purpose of the study	Conclusion/result
Becker et al. 2006 [4]	RCT	Effect of smoking cessation and its resumption on inhaled insulin	Smoking cessation for 1-2 weeks brings absorptions levels comparable to healthy non-smoker levels. Resumption for only 1-2 days can revert the changes back to chronic smoker levels.
Takano et al. 2012 [6]	Clinical experiment	Impact of cigarette smoke extract on insulin transport in alveolar epithelial cell line A549	Cigarette smoke extract directs cultured A549 cells to a type-II like phenotype, which enhances insulin uptake and overall increase of its transfer across the epithelial cell layer.
Himmelman et al. 2003 [8]	RCT	The impact of smoking on inhaled insulin	Higher absorption of inhaled insulin in smokers with higher AUC (0-6 hours), Cmax and shorter Tmax.
Wise et al. 2006 [15]	RCT	Effect of smoking on absorption and glucodynamic effects on inhaled insulin using the Lily-Dura inhaled insulin system	Smoking enhances the absorption of inhaled insulin but the utilization of glucose is not high, possibly due to a decrease in insulin sensitivity caused by smoking.
Pan et al. 2007 [48]	RCT	Effects of smoking cessation, acute re-exposure, and nicotine replacement in smokers on pharmacokinetics and glucodynamics of inhaled insulin	Smoking cessation for four weeks is associated with increased insulin exposure and glucodynamic response with its magnitude being higher in those subjects who are on nicotine replacement therapy.
Fountaine et al. 2008 [49]		Impact of acute-passive cigarette smoke exposure on inhaled insulin	Passive smoke reduces lung permeability and decreases the bioavailability of inhaled insulin.

The impact of smoking on inhaled insulin is well evident. Through many different ways, including permeability, epithelial cell changes, oxidative stress, smoking enhances or reduces insulin absorption depending upon the variability of the pattern (acute or chronic), patient characteristics. Himmelman et al. showed increased absorption in smokers and the increased impact of acute smoke compared to non-acute smoke. Wise et al. confirmed the same but also postulated that absorption of subcutaneous insulin also increased. However, there have been studies contradictory to this fact, which show decreased absorption of subcutaneous insulin after smoking due to cutaneous vasoconstriction caused by nicotine present in smoke [39, 46, 47]. However, there is no conclusive evidence for either of the facts. Wise et al. further mentioned that the effects of increased insulin bioavailability were not pronounced, probably due to decreased insulin sensitivity caused by CS, which has been put forward by many other studies as well.

Becker et al. also showed increased absorption and highlighted new information regarding the initial inhibitory of acute smoke. These findings are contradictory to the theory of increased permeability. Still, they instead likely could be because of hyperresponsiveness of the bronchial tree to the cigarette smoke and hence the initial lag in insulin absorption. However, this needs further clarification. The effect of smoking varies significantly with the type of smoking, that is acute or chronic or acute passive. The studies have all been done on non-diabetic patients, and over a short term, hence the generalisability will be an issue. We need more data on the long term effects. Smoking also causes insulin resistance and reduces its glucodynamic effects. Does this imply that with proper dosage and a better understanding of these factors, the use of inhaled insulin in smokers could be possibly contradictory to the common notion of not prescribing the inhaled insulin to smokers? The risk of hypoglycemia due to passive smoking is very low since passive smoke reduces the permeability. It is thereby highlighting another fact that demands more research on the role of bronchial reactivity to smoke, and its impact on inhaled insulin. Changes in airway reactivity seem to be more explanatory than permeability changes of passive smoke; the same can be true for acute active smoking.

**Figure 4:**
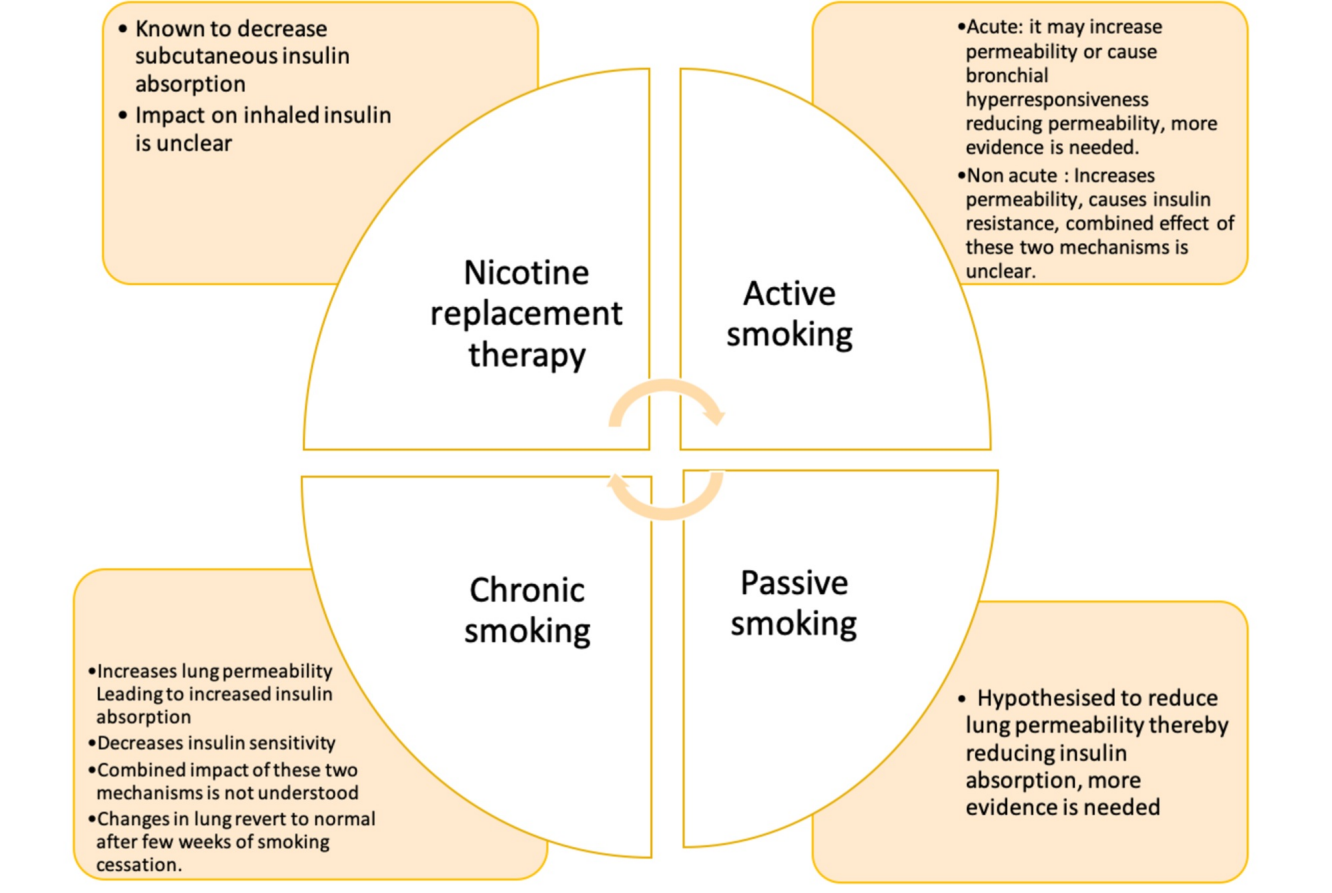
Summary of the impact of smoking on inhaled insulin

Further, not much data is available on nicotine's role in the pharmacokinetics or bioavailability of inhaled insulin. One study pointed out the impact of nicotine replacement therapy (NRT). We believe that nicotine replacement therapy's impact would be as significant as the impact of smoking on inhaled insulin since in abstinence from smoking, patients are put on NRT. Mohammed et al. have published their data on nursing considerations associated with inhaled insulin, which highlighted the fact that since diabetes is a chronic condition, long term studies with various variables need to be conducted [2]. The following Figure 4, concludes the impact of smoking on inhaled insulin and the potential areas of further exploration. It summarises the hypothesized mechanisms and highlights the unclear aspects.

Our study combines the available data from the 1970s until 2020 on smoking's impact on inhaled insulin. It does not include all the studies on inhaled insulin. An attempt has been made to explain the effect of both smoking and inhaled insulin on the lungs at a molecular level. Still, only the data on the role of smoking on the epithelial layer has been highlighted since the epithelial layer is the source of absorption for inhaled insulin. The most recent updates on the development of inhaled insulin devices have not been mentioned. The article focuses on a very specific aspect of inhaled insulin, however, there are many more factors that influence its use. A combined effect of all the factors would play a role to determine its use in patients.

## Conclusions

Diabetes is a chronic illness and needs long term treatment in the form of oral drugs or subcutaneous injections. For those on subcutaneous injections, inhaled insulin could be a good alternative that would most importantly improve patient compliance and result in better glycemic control. Active smoking increases the permeability of inhaled insulin, passive smoking has an opposite effect, although more clarifications are needed on the same. An increase in insulin resistance, caused by smoking, will also affect the overall impact and hence the usage of inhaled insulin. Since smoking is highly prevalent in diabetes and shares the same target organ as inhaled insulin, it should be researched for the long term in diabetic patients or a more generalizable population sample. This can also be an excellent opportunity to make smokers leave their habit and adopt a healthier lifestyle .
